# Structural and Functional Characteristics of Two Molecular Variants of the Nitrogen Sensor PII in Maritime Pine

**DOI:** 10.3389/fpls.2020.00823

**Published:** 2020-06-16

**Authors:** María Teresa Llebrés, María Belén Pascual, Carolina Valle, Fernando N. de la Torre, José Miguel Valderrama-Martin, Luis Gómez, Concepción Avila, Francisco M. Cánovas

**Affiliations:** ^1^Grupo de Biología Molecular y Biotecnología de Plantas, Departamento de Biología Molecular y Bioquímica, Faculty of Science, Universidad de Málaga, Campus Universitario de Teatinos, Málaga, Spain; ^2^Centro de Biotecnología y Genómica de Plantas, Campus de Excelencia Internacional de Montegancedo, Parque Científico y Tecnológico de la Universidad Politécnica de Madrid, Madrid, Spain

**Keywords:** *Pinus pinaster*, amino acids, arginine metabolism, isoproteins, nitrogen storage

## Abstract

High levels of nitrogen are stored as arginine during the last stages of seed formation in maritime pine (*Pinus pinaster* Aiton). The protein sensor PII regulates the feedback inhibition of arginine biosynthesis through interaction with the key enzyme *N*-acetylglutamate kinase (NAGK). In this study, the structural and functional characteristics of PII have been investigated in maritime pine to get insights into the regulation of arginine metabolism. Two different forms of PII have been identified, PpPIIa and PpPIIb, which differ in their amino acid sequence and most likely correspond to splicing variants of a single gene in the pine genome. Two PII variants are also present in other pine species but not in other conifers such as spruces. PpPIIa and PpPIIb are trimeric proteins for which structural modeling predicts similar tridimensional protein core structures. Both are located in the chloroplast, where the PII-target enzyme PpNAGK is also found. PpPIIa, PpPIIb, and PpNAGK have been recombinantly produced to investigate the formation of NAGK-PII complexes. The interaction of PpPIIa/PpPIIb and PpNAGK may be enhanced by glutamine and contribute to relieve the feedback inhibition of PpNAGK by arginine. Expression analysis of *PpPII* genes revealed that PpIIa transcripts were predominant during embryogenesis and germination. The potential roles of PpPIIa and PpPIIb in the regulation of arginine metabolism of maritime pine are discussed.

## Introduction

Plants, like other organisms, have developed mechanisms to detect and respond to changes in carbon and nitrogen levels. These mechanisms regulate the activity of proteins involved in the transport and metabolism of nitrogen and carbon compounds, allowing plants to optimize their energy sources. These processes are particularly important in trees due to their long life cycles (Cánovas et al., 2018).

The sensing mechanisms for carbon/nitrogen (C/N) balance activate genes involved in nitrogen assimilation when carbon skeletons are abundant and internal levels of organic nitrogen are low. On the other hand, they decrease the rate of nitrogen assimilation when the levels of photosynthetic products are low or the internal levels of nitrogen are relatively high. These mechanisms allow plants to adjust their energy and metabolite allocation to a shifting carbon and nitrogen availability (Coruzzi and Zhou, 2001; Gent and Forde, 2017).

The systems to sense and respond to carbon and nitrogen levels have been the subject of elegant studies on microorganisms such as *Escherichia coli* and yeast (Forchhammer and Lüddecke, 2016). In these unicellular models, signaling systems have been found to be complex and multiple (Jiang et al., 1998). These systems are even more complex in plants, which must respond to various carbon and nitrogen metabolites in different tissues, stages of development, and cell compartments (Ferrario-Méry et al., 2006, 2008). A key protein family of modulators acting in microorganisms and plants is the one of the PII signaling proteins (Selim et al., 2020a). These proteins have as targets channels, enzymes, and transcriptional regulators, influencing the activities of these targets by direct protein–protein interactions with them (Selim et al., 2020a). PII proteins sense the nitrogen, carbon, and energy richness status of the cell via interactions with allosteric effectors [ATP/ADP and 2-oxoglutarate (2OG); and, in plants, also with glutamine] and, in some bacteria, by highly specific postranslational modifications (Forchhammer and Lüddecke, 2016). In this way, PII proteins are signal transductors that play crucial roles for nitrogen/carbon and energy regulation of bacteria and also of plants. They are the main focus of the studies presented here.

PII proteins are homotrimers of a subunit presenting a well-conserved sequence which is folded according to the ferredoxin fold. This fold is made of two βαβ structural repeats, and in PII proteins, it presents three functionally important loops, called T-loop, B-loop, and a C-terminal loop. The T-loop hosts a characteristic invariant sequence motif (Motif I). This loop connects both repeats of the fold, it is long and plastic, and its conformation is influenced by the allosteric effectors of PII, also being in some bacteria the site of covalent modifications at specific residues (as exemplified by the T-loop uridylylation observed in *E. coli*, Ninfa and Atkinson, 2000). The T-loop plays a key role in the interactions of PII with its protein partners (Selim et al., 2020a). The B-loop also hosts an invariant motif (Motif II) and also participates in the interactions with PII targets (Selim et al., 2020a). The C-terminal loop of plants is called the Q-loop, it is longer than bacterial C-terminal loops, and it is the site of glutamine binding. The adenine nucleotide and 2OG effectors sit at intersubunit crevices at the root of the T-loops.

In bacteria, the signaling PII protein serves as a central processing unit to integrate signals on carbon, nitrogen, and energy abundance status, and use such information to control nitrogen uptake (Ninfa and Atkinson, 2000). The assimilation of ammonium as glutamine is regulated in response to intracellular concentrations of glutamine (a nitrogen signal) and 2-OG (a carbon signal) (Muro-Pastor et al., 2005; Robles-Rengel et al., 2019).

In plants, the PII protein is a sensor of C/N status mediated by the binding to 2-OG and glutamine. It subsequently regulates the arginine biosynthetic pathway (Chen et al., 2006; Chellamuthu et al., 2014) which is under feed-back control by arginine inhibition of *N*-acetylglutamate kinase (NAGK). However, when N is abundant, the inhibition is relieved through the interaction of NAGK with the sensor protein PII (Chen et al., 2006; Llácer et al., 2007). Increased levels of glutamine are a signal of N abundance that is sensed via the PII Q-loop and transduced into enhanced activity of NAGK (Chellamuthu et al., 2014). In this way, the connection proposed between PII and arginine-rich protein biosynthesis and deposition in seeds (Urigh et al., 2009) could be regulated by glutamine availability. Other less well-studied effects of PII on plant nitrogen metabolism include the observation made that in symbiotic nitrogen fixation PII affects the development of root nodules, the mobilization to the host of of the fixed nitrogen, and the signaling of the N nutritional status (Arcondeguy et al., 2001; D’Apuzzo et al., 2015), as well as the recent report of a potential role of PII in the biosynthetic production of NO (Parlatti et al., 2017).

In trees, when the availability of N is high, arginine is stored either as a free amino acid or as a component of storage proteins in the bark and seeds (Li and Coleman, 2019). During embryogenesis, nitrogen derived from tissue sources, mainly in the form of glutamine, is transported to developing seeds where it is used for the biosynthesis and deposition of storage proteins. In conifers, arginine is highly abundant in the seed storage proteins and represents an important source of nitrogen for developing plantlets following seed germination (Todd et al., 2001; Todd and Gifford, 2003; Cánovas et al., 2007). Therefore, the regulation of arginine metabolism has a relevant role in the store and mobilization of N during the embryogenesis and germination of pine (Llebrés et al., 2018a, b).

*In vitro* somatic embryogenesis in combination with cryopreservation is a major biotechnological tool for vegetative propagation of selected maritime pine varieties for different ecological and economical applications (Trontin et al., 2016). However, a fundamental understanding of how arginine metabolism is regulated during embryogenesis and germination is necessary to improve embryo quality and to generate vigorous maritime pine seedlings via somatic embryogenesis (Llebrés et al., 2018b).

In the present study, key aspects of PII regulation in maritime pine are investigated with the purpose of getting insights into the regulation of arginine biosynthesis in response to nitrogen availability in conifers, a plant group of crucial ecological and economic importance.

## Materials and Methods

### Plant Material

Maritime pine embryos were excised from seeds collected from a single seed orchard (Picard, Saint-Laurent-Médoc, France) at different developmental stages (Llebrés et al., 2018b). *Pinus pinaster* Aiton seeds were provided by the Centro Nacional de Recursos Genéticos Forestales from “Ministerio de Agricultura, Pesca, Alimentación y Medio Ambiente,” Spain. Seeds were imbibed in distilled water for 24 h under continuous aeration and germinated and grown with vermiculite as a substrate under a photoperiod of 16 h light/8 h dark at 24°C. Hypocotyls of pine seedlings were collected after 2 weeks, frozen in liquid nitrogen, and stored at −80°C until use. *Nicotiana benthamiana* L. seeds were sown and grown in pots and maintained under a 16 h light/8 h dark photoperiod at 24°C for 5 weeks.

### Cloning of PpPIIa, PpPIIb, and PpNAGK

The sequences of *PpPIIa*, *PpPIIb*, and *PpNAGK* were retrieved from the maritime pine (*P. pinaster*) transcriptome available at SustainPineDB v.3.0^1^. Full-length cDNAs were isolated from pine hypocotyl RNAs by reverse transcription-PCR (RT-PCR) using primers designed from *P. pinaster* sequences (Canales et al., 2014). Extraction of RNA was performed as described by Canales et al. (2012) and quantified using a NanoDrop© ND-1000 spectrophotometer. Synthesis of cDNA was performed with 5X iScript^TM^ cDNA Synthesis Kit (Bio-Rad). The primer pairs used for specific amplification are listed in Supplementary Table S1. The resulting PCR products were cloned in a pJet1.2 (Thermo Fisher Scientific^TM^) vector and completely sequenced.

### Transient Expression of GFP-Proteins in *Nicotiana benthamiana*

PCR amplifications were performed as described above but the stop codons were removed from reverse primer sequences (Supplementary Table S1). The resulting PCR products were cloned into pDONR207 and subcloned into pGWB5 via Gateway technology (Invitrogen) to produce, under the control of the CaMV *35S* promoter, full-length proteins fused to GFP at their C-termini (El-Azaz et al., 2016). Empty pGWB5 was used for negative controls. The *Agrobacterium tumefaciens* strain C58C1 was transformed by electroporation with recombinant plasmids. *N. benthamiana* leaves (5 weeks old) were syringe infiltrated with cultures containing pGWB5 constructs mixed with cultures containing the silencing suppressor p19 protein, both with an optical density of 0.5 at 600 nm, according to previously described procedures (Liu et al., 2002). Subcellular localization of proteins was examined by confocal microscopy 36–48 h after agroinfiltration.

### Transient Expression of GFP-Proteins in Pine Protoplasts

To perform transient expression assays in pine protoplasts, fusions of PpPIIa with or without its putative chloroplastic transit peptide (cTP) and the GFP reporter gene were constructed using appropriate primers. Protoplasts were prepared from maritime pine cotyledons by incubation of 1 g of freshly cutted tissue in 10 mL of a mixture containing 0.44% (w/v) K3 medium: 0.4% (w/v) cellulase, 0.4% (w/v) macerase (Calbiochem), and 0.4% sucrose. Transformation with the gene constructs was performed by electroporation essentially as previously described by Gómez-Maldonado et al. (2004). After culture in the dark for 18–24 h, the transformed protoplasts were visualized by laser confocal microscopy using a Leica CLSM microscope. Excitation was conducted with a laser beam at 488 nm. Red autofluorescence of chlorophyll was detected up to 560 nm and green fluorescence of GFP was detected between 505 and 520 nm.

### Overexpression and Purification of Recombinant Proteins

Open reading frames (ORFs) corresponding to *PpPIIa* and *PpPIIb* without the cTP were PCR-amplified using primers containing appropriate restriction sites. *PpPIIa* and *PpPIIb* forward primers contained a *NdeI* restriction site and the reverse primer contain a *XhoI* restriction site. Sequences are listed in Supplementary Table S1. PCR products were digested and subcloned into the pET30b at *Nde*I and *Xho*I sites. Plasmid constructs were sequenced and subsequently transformed into *E. coli* strain BL21-AI (Thermo Fisher Scientific). Cells were grown at 37°C by shaking in Luria–Bertani broth containing 50 μg mL^–1^ kanamycin until OD_600_ = 0.6; 0.2% arabinose (w/v) was added, and cultures were further incubated for 5 h at 30°C with shaking. Cells were pelleted by centrifugation at 4,500 × *g* and frozen. ORF corresponding to *PpNAGK* without the cTP was amplified using a specific forward primer (Supplementary Table S1). The resulting PCR product was cloned into the pDONR207, subcloned into the pDest17 vector via Gateway Technology (Invitrogen), and transformed into *E. coli* strain BL21-AI (Thermo Fisher Scientific). Bacterial growth and protein induction requirements were the same as described above but the media contained 100 μg mL^–1^ ampicillin instead. Recombinant proteins were purified by binding onto Ni-agarose resin (Protino NiNTA; Macherey-Nagel). Protein concentrations were determined by the Bradford dye-binding method (Bradford, 1976) and analyzed by SDS-PAGE to verify purification.

### PpNAGK Activity Assays

The activity assay based on *N*-acetylglutamylhydroxamate production was performed according to the method described by Heinrich et al. (2004). The reaction mixture consisted of 400 mM NH_2_OH-HCl, pH 7, 20 mM Tris/Cl, pH 7, 20 mM MgCl_2_, 40 mM NAG, and 10 mM ATP. Reactions were initiated by the addition of reaction mixture, the incubation was carried out at 37°C in a volume of 150 μL and terminated by the addition of 150 μL of stop mixture [1:1:1 of 5% (w/v) FeCl_3_ ⋅ 6 H_2_O in 0.2 M HCl, 8% (w/v) trichloroacetic acid, and 0.3 M HCl]. After standing for 5 min at room temperature, the tubes were centrifuged and the absorbance was immediately measured at 540 nm. The absorbance change was linear with time of incubation in the enzyme assay for at least 30 min. Controls were carried out that did not contain *N*-acetylglutamate. There was no activity when no NAGK was added. To estimate the molar amount of *N*-acetylglutamylhydroxamate formed a molar absorption coefficient was used of 456 M^–1^cm^–1^ at 540 nm (Heinrich et al., 2004).

*N*-acetylglutamate kinase activity was also determined by a continuous spectrophotometric assay in which the production of ADP was coupled to NADH oxidation via pyruvate kinase (PK) and lactate dehydrogenase (LDH) (Cánovas et al., 1984). The reaction mixture containing 100 mM Hepes, pH7, 10 mM MgCl_2_, 10 mM ATP, 40 mM *N*-acetylglutamate, 1 mM phosphoenolpyruvate, 0.6 mM NADH, and 1 unit each of PK and LDH. Assays were performed in 100 μL volumes and incubated at 37°C in multi-well plates for 5 min. The blank reactions did not contain *N*-acetylglutamate.

### Gel Filtration Chromatography of Purified PII Proteins

Protein samples were diluted to a concentration of 0.6 μg/μL in a solution of 25 mM Tris-HCl, pH 7.5, 150 mM NaCl, 10% (v/v) glicerol, and a 250 μL sample was then chromatographed on a ENrich^TM^ SEC 650 10 × 300 column (BioRad) equilibrated at room temperature in Tris/HCl buffer. The fast protein liquid chromatography flow rate was 0.75 mL/min, and 100 μL fractions were collected.

The column was calibrated with ribonuclease A (13.7 kDa), carbonic anhydrase (29 kDa), bovine serum albumin (66 kDa), alcohol dehydrogenase (150 kDa), ß-amylase (200 kDa), apoferritin (440 kDa), and blue dextran (void volume of the column).

### Western Blot and Antibody Production

The presence of proteins in the elution fractions was detected by SDS-PAGE and western blot analysis with anti-PII antibodies as described previously (Cánovas et al., 1991). Antibodies against PpPIIa were raised in rabbits by immunization with PpPIIa recombinant protein overexpressed in *E. coli* as described by Cantón et al. (1996). The primary antibodies anti-PpPIIa were used at 1:10,000 dilution and a 1:10,000 dilution of horseradish peroxidase conjugated antirabbit serum (Sigma) was used as a secondary antibody.

### Yeast Two-Hybrid Assay

Interaction assays in yeast were performed with the yeast Two-Hybrid System (YTH) of Invitrogen. PpNAGK was cloned into Gateway pDEST22 vector by LR recombination reaction to form the prey plasmid, whereas PpPIIa and PpPIIb were cloned into Gateway pDEST32 vector by LR recombination reaction to form the bait plasmids. Two mutations were produced in PpPIIa corresponding to a single change of a glutamate for lysine (Glu18Lys) and a triple amino acid change: valine, alanine, and glycine, for alanine, glycine, and alanine (Val58Ala/Ala60Gly/Gly61Ala). Site-directed mutagenesis was performed through a PCR-based strategy using iProof high-fidelity DNA polymerase (Bio-Rad) and cloned into pDEST32 vector. Specific primers were used for amplification under the following conditions: 2 min at 98°C, followed by 20 cycles (10 s at 98°C, 20 s at 60°C, and 7 min at 72°C) and a final elongation of 4 min at 72°C. Samples were treated with *Dpn*I (Thermo Scientific^TM^) to eliminate native methylated plasmid DNA, and the PCR product was transformed into *E. coli* DH5α with selection on 100 μg mL^–1^ gentamicin. All of the constructs were verified by sequencing and co-transformed into yeast stain MAV203 using the LiAc method following the yeast transformation instructions of Invitrogen. Transformed yeasts were selected at 30°C on synthetic defined (SD) media agar plates without leucine and tryptophan.

To identify strong interactions, individual transformants were characterized on SD agar plates without leucine, tryptophan, and histidine, supplemented with 10, 25, 50, and 100 mM 3-AT (3-Amino-1, 2, 4-triazole, Sigma) and on SD agar plates without leucine, tryptophan, and uracil.

Positive colonies were grown in liquid media to the stationary phase. Aliquots of 1 mL were taken and cells were pelleted at 11,000 × *g* for 30 s, the media was removed and the cells were then washed five times with sterile distilled water (SDW). Cells were then resuspended in SDW and diluted 10, 100, and 1000 times. A volume of 10 μL from each sample was spotted onto plates containing SD media without leucine, tryptophan, and uracil. Plates were incubated at 30°C for 3 days and photographed.

### Quantitative β-Galactosidase Assay

Strong YTH interactions of PpNAGK with PpPIIa and PpPIIb were quantified by the liquid β-galactosidase assay with ONPG (*o*-nitrophenyl β-D-galactopyranoside; Sigma Cat No. N-1127), following the Yeast Protocols Handbook user manual from Clontech Laboratories. All measurements were made in triplicate.

### RNA Extraction and Expression Analysis

RNA extraction from maritime pine samples was performed as described by Canales et al. (2012). Synthesis of cDNA was made with 5X iScript^TM^ cDNA Synthesis Kit (Bio-Rad). The qPCR analysis was done in a thermal cycler CFX384 (Bio-Rad). Sequences of *PpPIIa* and *PpPIIb* specific primers are listed in Supplementary Table S1. Actin-7 was used as a reference gene. Relative expression profiles for each gene were obtained employing the R package (Ritz and Spiess, 2008) and normalized to the reference gene. For the qPCR analysis, three biological replicates and three technical replicates per sample were made.

### Structural Modeling and Calculations

Structural models for PpPIIa and PpPIIb were generated with the Swiss-Model Server (Biasini et al., 2014) using as template the structure of the *Chlamydomonas* PII protein as observed in its crystal structure in complex with the NAGK from *Arabidopsis thaliana* (PDB entry 4USJ, chain C). Alternative models were generated with the I-TASSER suite (Yang et al., 2015), from which the geometry of the first 10 residues was predicted. Energy minimization and structural superpositions were performed with the 1.11.2 version of the UCSF Chimera package^2^ (Pettersen et al., 2004). The electrostatic Poisson–Boltzmann (PB) potential was deduced with the 300 APBS tools in PyMol 1.7.6.7 (Schrödinger, LLC) assigning AMBER atomic charges and radii. The non-linear PB equation was solved in sequential multigrid calculations at 298 K with dielectric constants of 2 for proteins and 78.54 for water. Values of potential are given in kT units per unit charge (k, Boltzmann’s constant; T, absolute temperature). PB potentials mapped onto protein surfaces were rendered with PyMOL. Other figures were rendered with UCSF Chimera.

## Results

### *In silico* Profile of Maritime Pine PII

Recent developments in next generation sequencing have provided a range of genomic resources for maritime pine (Canales et al., 2014; Cañas et al., 2017, 2019). The *in silico* analysis of its transcriptome revealed the existence of two different transcripts encoding putative PII proteins that were named *PpPIIa* and *PpPIIb* (Supplementary Figure S1). A striking characteristic of these transcripts is that their sequences mostly differ in the 5’-unstranslated region and the ORF, with minor changes in the 3’-unstranslated region (Supplementary Figure S1).

**FIGURE 1:**
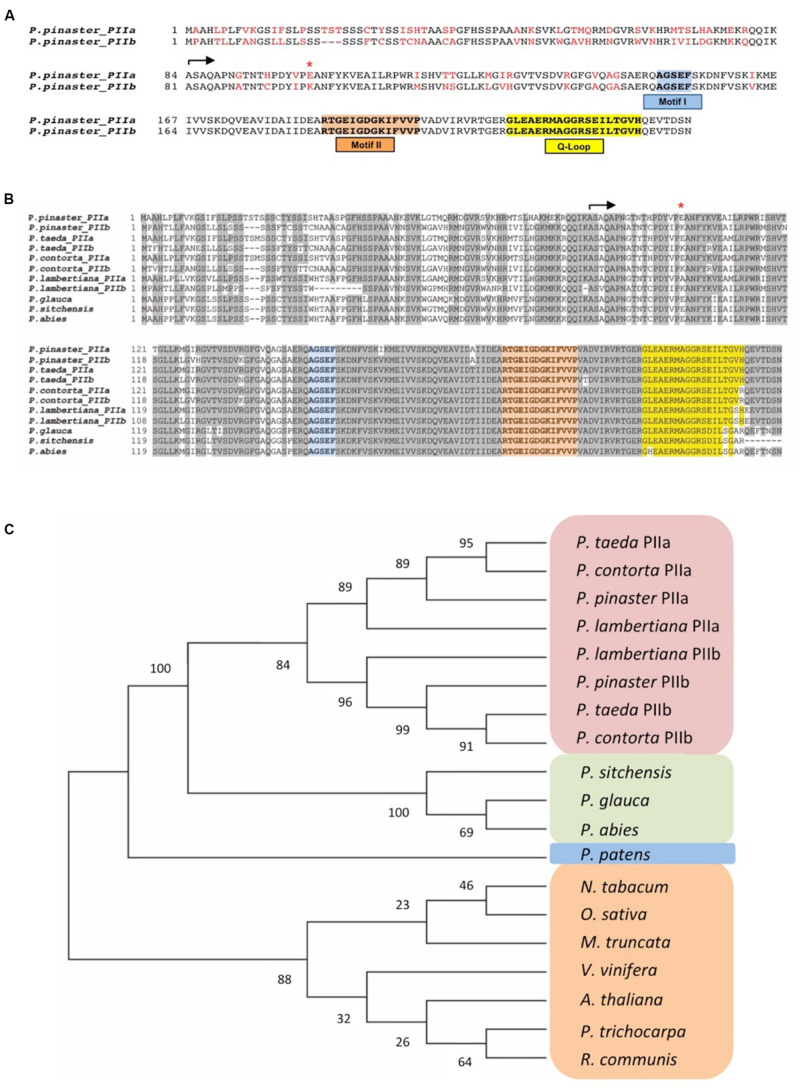
Comparison of PII sequences. **(A)** Predicted PpPII protein sequences. The conserved sequence motifs (I and II) and the Q Loop position are boxed. The beginning of both mature polypeptides is indicated by an arrow. Residues differing in the two variants are marked in red. The red asterisk highlights a significant amino acid change at position 18 of the mature proteins (see text). **(B)** Multiple alignments of PII proteins from pine species executed with Clustal X. Conserved sequence motifs in PII proteins are highlighted: in blue Motif I, in orange Motif II, and in yellow the conserved Q-loop of plants. **(C)** Phylogenetic analysis of PII proteins in angiosperm and gymnosperm species. Alignments were imported into the Molecular Evolutionary Genetics Analyses (MEGA) software version 7.0 (Kumar et al., 2016). The phylogenetic tree was constructed with the full-length PII amino acid sequences using the neighbor-joining method. Protein sequences were obtained from the following databases: NCBI (www.ncbi.nlm.nih.gov), PlantGDB (www.plantgdb.org), TAIR (https://www.arabidopsis.org), SustainPineDB v.3.0 (http://www.scbi.uma.es/sustainpinedb/sessions/new), and PLAZA (https://bioinformatics.psb.ugent.be/plaza/versions/gymno-plaza).

The ORFs of *PpPIIa* and *PpPIIb* encode polypeptides of similar size, with PpPIIa being three amino acids longer (Supplementary Table S2). Upon removal of the putative cTP, mature PpIIa and PpIIb proteins would have equal number of amino acids although their sequences differ in 15 amino acid residues (∼90% identity), with all the amino acid changes spread over the first half of the amino acid sequence of the mature putative proteins (Figure 1). Whereas most changes are conservative, a substantial change of glutamate for lysine was identified at position 18 of the mature proteins (Figure 1A, asterisk). Since only a single form of PII has been previously described in angiosperms, the occurrence of PII variants was further investigated in those conifers for which transcriptomic data are available. In addition to *P. pinaster*, several pine species contain two PII variants, including *Pinus taeda*, *Pinus contorta*, and *Pinus lambertiana*. However, only one PII form was found in spruce species (Figure 1B). Their amino acid sequences were highly conserved in pine including the aforementioned non-conservative shift at position 18. The phylogenetic analysis of PII protein variants clearly classified the pine proteins in two separate clusters, with the PIIb cluster being closer to PII proteins from other conifer species that represent an intermediate branch between pines and herbaceous plants (Figure 1C).

### Localization of Maritime Pine PpPII

**FIGURE 2:**
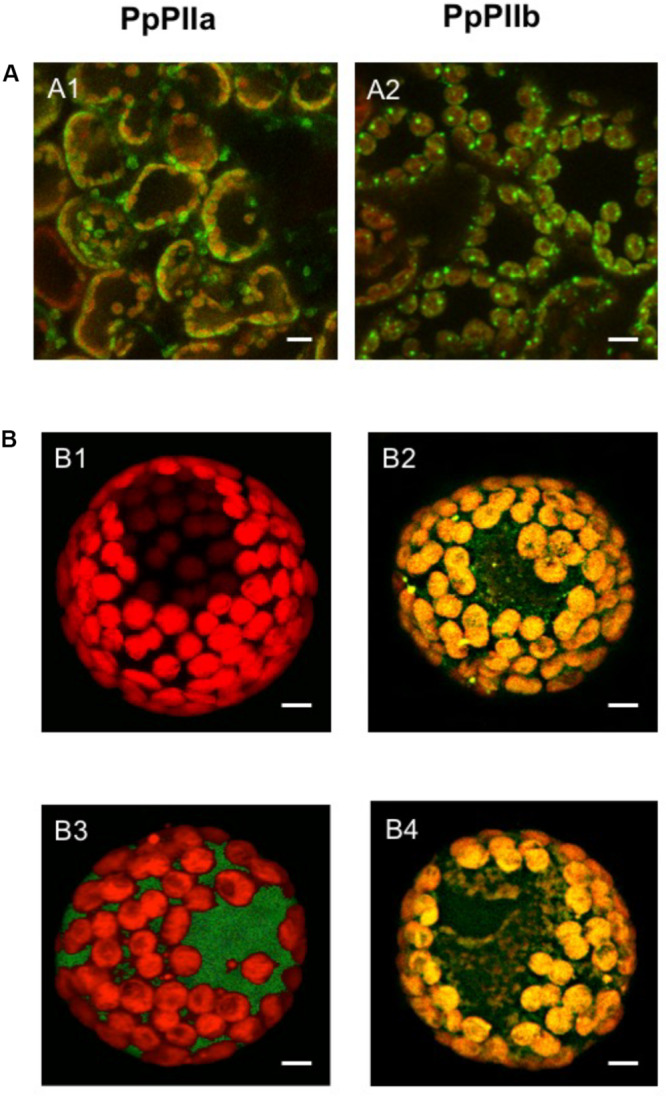
Subcellular localization of PpPIIa and PpPIIb. **(A)** Localization in *N. benthamiana* leaves. **(A1,A2)** GFP-chlorophyll merged images showing respective localizations in the chloroplast of PpPIIa and PpPIIb. **(B)** Localization in isolated pine protoplasts. A single cell is shown containing plastids. **(B1)** control with no construct; **(B2)** GFP–PpPIIa construct containing transit peptide (cTP); **(B3)** GFP-PpPIIa construct without cTP; **(B4)** GFP-control construct without the PpPIIa protein but containing cTP. Scale bar represents 10 μm.

To further understand the biological roles of these proteins, their subcellular localization was determined (Figure 2). The ORFs of PpPIIa and PpPIIb were PCR-amplified and cloned into the Gateway vector pGWB5 and the resulting GFP fusions were transiently expressed in *N. benthamiana* leaves via agroinfiltration. The GFP fluorescence of both PpPIIa and PpPIIb fusion products was exclusively associated to chloroplasts, which were visualized by the red autofluorescence of chlorophyll. The merged images showed co-localization of chlorophyll (red) with both GFP–PpPIIa and GFP–PpPIIb (yellow) in the chloroplasts, indicating a plastidic localization for both proteins (Figures 2A1,A2).

To further substantiate this finding, GFP fusions for PpPIIa were constructed with and without cTP and electroporated into isolated maritime pine protoplasts. Figure 2B shows that GFP–PpPIIa constructs containing the cTP were appropriately targeted to the chloroplasts (Figure 2B2), as it was also observed for control constructs with cTP but lacking the PpPIIa coding sequence (Figure 2B4). However, only cytosolic GFP signals were observed for the constructs lacking cTP (Figure 2B3).

### Structural Models of PpPIIa and PpPIIb

**FIGURE 3:**
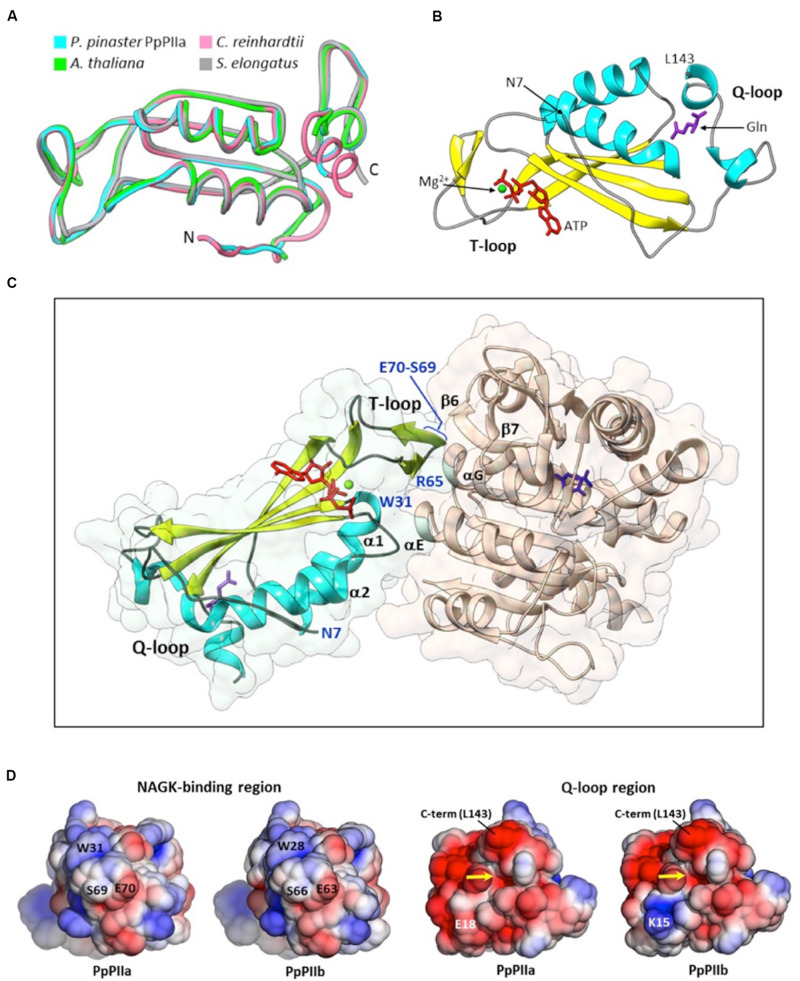
Model structures for PpPIIa and PpPIIb. **(A)** Structural alignment of modeled PpPIIa and the experimentally solved structures for AtPII (PDB entry 2RD5), SePII (PDB entry 2V5H), and CrPII (PDB entry 4USJ), three homologous proteins crystallized in complex with NAGK and various ligands. **(B)** Cartoon representation of PpPIIa, highlighting the position of the N-terminal extension, the T-loop and the Q-loop. The position of L143 (PpPIIa numbering; C-terminal end of the model) is also indicated. Docked ligands: Mg^2+^ (green), ATP (red), and glutamine (purple). Compared to **(A)**, this view is rotated about 90° upward. **(C)** Side view of a dimer between PpPIIa and/**/NAGK from *A. thaliana* (PDB code 2RD5). The PII residues directly involved in interactions with the *Arabidopsis* enzyme are highlighted in blue, like the N-terminus of the model (N7). A molecule of *N*-acetylglutamate is shown at the active site of AtNAGK (dark blue). **(D)** Electrostatic potential distribution mapped to the solvent-accessible surfaces of PIIa and PIIb at two opposed ends: the region for NAGK binding (left pair) and the confluence of the N-terminal extension and the Q-loop (right pair). The glutamine-binding site is indicated by a yellow arrow. The potential is shown as a colored gradient from red (acidic) at -2.5 kT/e to blue (basic) at 2.5 kT/e (where k is Boltzmann’s constant, T is temperature, and e is the charge on an electron).

To gain insight into the functional significance of the amino acid differences found between mature PpPIIa and PpPIIb, their three-dimensional structures were modeled with the Swiss-Model server using as template the reported structure of Chlamydomonas PII (taken from PDB entry 4USJ, see section “Materials and Methods”), as well as by using I-TASSER, which chooses automatically model structures from the PDB database. Despite the different approaches and models used by these servers, both servers predicted essentially the same geometries for the protein cores (RMSD of 0.63–0.64 Å). The overall fold (137 residues) was remarkably similar to the X-ray structures of PII proteins previously crystallized in complex with NAGK, such as *A. thaliana* PII (AtPII; PDB entry 2RD5; Mizuno et al., 2007), *Synechococcus elongatus* PII (SePII; PDB entry 2V5H; Llácer et al., 2007), and *Chlamydomonas reinhardtii* (CrPII; PDB entry 4USJ; Chellamuthu et al., 2014) (Figure 3A). CrPII was the template we selected for Swiss-Model, to obtain atomic coordinates for the Q-loop residues (see below). The structural alignment of these experimentally solved proteins with PpPIIa rendered RMSD values as low as 0.29 Å (CrPII; 132 Cα pairs), 0.67 Å (AtPII; 127 Cα pairs), and 0.70 Å (105 Cα pairs), bolstering the validity of the predicted geometries. PpPIIa and PpPIIb appeared to contain all the secondary structure elements distinctive of PII proteins (reviewed by Forchhammer and Lüddecke, 2016), including the T-loop involved in Mg^2+^. ATP binding and the C-terminal Q-loop for glutamine sensing (Figure 3B). PII proteins from cyanobacteria and Brassicaceae lack a glutamine binding site at the C-terminal part of the polypeptide (SePII and AtPII in Figure 3A). The conserved spatial positions of key residues for ligand binding allowed us to dock Mg^2+^. ATP in the T-loop of both models, and the same is true for glutamine in the Q-loop (Figure 3B). Based on the known architectures for PII-NAGK complexes, we also analyzed the putative contact region with NAGK. Specific residues of the pine PIIs can be predicted to stabilize the heterodimer by interacting with complementary groups located near the end of helices αE and αG of NAGK as well as in the neighboring strands β6 and β7 (Figure 3C). On the PII side, the key interacting residues were located at the end of the α1-helix (W31) and in the T-loop (mainly R65, S69, and E70; numbering for PpPIIa). More specifically, W31 would participate in both hydrogen bonding (backbone) and hydrophobic contacts, whereas the T-loop residues are predicted to form hydrogen bonds and electrostatic interactions with oppositely charged residues of NAGK. While the above structural analyses are consistent with a common mechanism of action for PpPIIa and PpPIIb, calculations of the PB electrostatic potential might explain the presence of two isoforms in maritime pine. We performed a thorough comparison of the electrostatic potential projected onto the surface of both proteins and did not find any significant difference for the regions involved in interacting with NAGK (Figure 3D, left) or PII trimerization (not shown). However, a significant effect was observed for the substitution of glutamate at position 18 of PpPIIa for lysine in PpPIIb. This notable change in the electrostatic features of the N-terminal extension (Mizuno et al., 2007) occurs in the vicinity of the Q-loop, not far from the glutamine-binding pocket (Figure 3D right). It has been suggested that such N-terminal extension, which maps on the opposite side of the trimer than the surface of interaction with NAGK, may be involved in protein–protein interactions and signaling (Mizuno et al., 2007; Selim et al., 2020a). Interestingly, most non-conservative mutations occur in this extension (see the alignments presented in Figure 1).

### Activity of the Complex PpNAGK–PpPII

The mature PII isoproteins from maritime pine were recombinantly expressed in *E. coli* to raise monospecific antibodies and for further characterization (Supplementary Figure S2). Moreover, sufficient amounts of recombinant native proteins were necessary to perform functional studies on the interaction of PpPIIa and PpPIIb with pine NAGK (PpNAGK) and to investigate how this interaction is regulated by the availability of glutamine and 2-OG.

**FIGURE 4:**
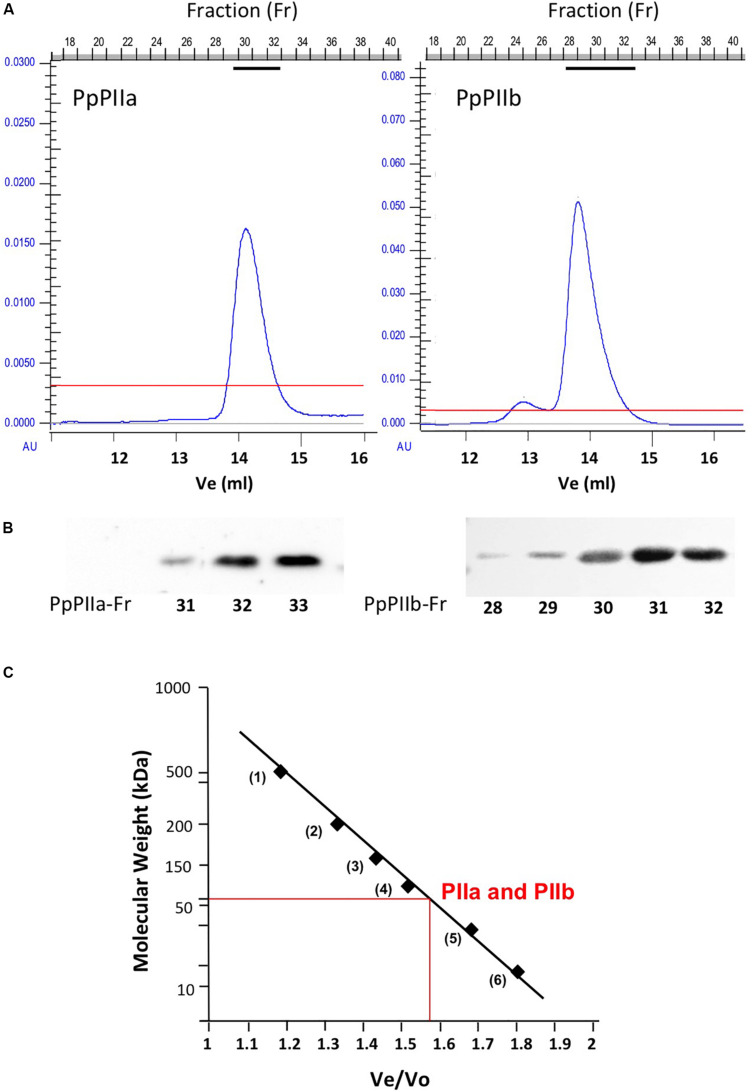
Estimation of the molecular size of native PpPII proteins by gel filtration chromatography. **(A)** Elution profile of recombinant PpPIIa and PpPIIb proteins using gel filtration in an FPLC system. **(B)** Protein-containing fractions were tested by western blotting using monospecific antibodies against PpPIIa. **(C)** Estimation of PpPIIa and PpPIIb molecular mass using protein markers of known molecular size.

The native molecular mass of PpPIIa and PpPIIb was determined by gel filtration chromatography through a FPLC column calibrated with protein standards (Figure 4). The molecular mass of both holoproteins was estimated to be 57 kDa, a figure compatible with a trimeric structure for the native proteins.

The target enzyme of maritime pine PpPII proteins is PpNAGK, a protein of 352 amino acids. The N-terminus of the predicted polypeptide targets the protein to plastids, where the mature enzyme has been recently localized (Llebrés et al., 2018b). To examine the potential differences in the interaction of PpPIIa and PpPIIb with their target enzyme, a full-length cDNA for PpNAGK was amplified using appropriate primers. A construct encoding the mature PpNAGK enzyme (301 residues without cTP) was fused to a His-tag and overexpressed in *E. coli.* The resulting recombinant protein was purified by affinity chromatography and next used for additional studies.

**FIGURE 5:**
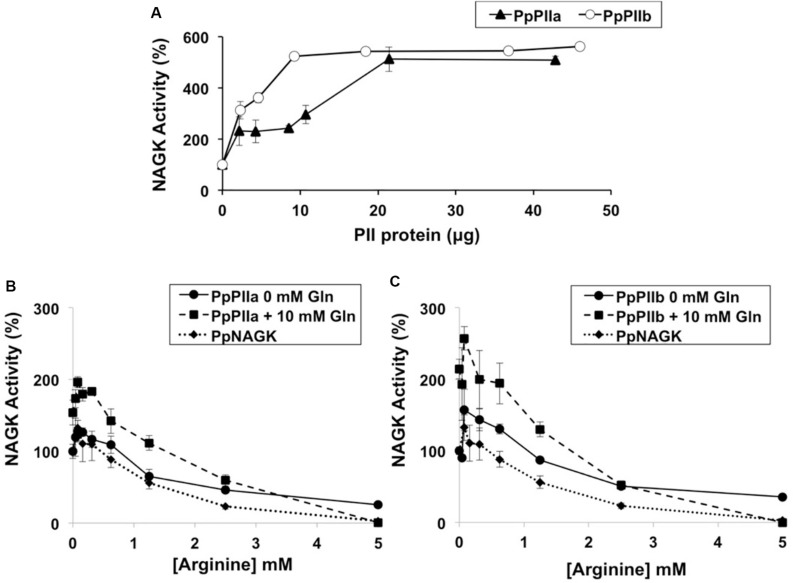
Effects of PpPIIa and PpPIIb on the PpNAGK activity. **(A)** Effect of increasing amount of PpPIIa (black triangles) and PpPIIb (open circles) proteins on PpNAGK activity. The indicated amounts of PpPII were mixed with 0.65 μg PpNAGK, and the mixtures were placed at 4°C for 10 min to allow complex formation. The reaction was initiated by the addition of the reaction mixture into the PpNAGK-PpPII complex mixture. The *N*-acetylglutamylhydroxamate assay was used. **(B)** PpNAGK–PpPIIa complex activity inhibition by arginine with or without added glutamine. **(C)** PpNAGK–PpPIIb complex activity inhibition by arginine with or without added glutamine. Glutamine 10 mM (black squares); glutamine 0 mM (black circles). PpNAGK activity inhibition by arginine is also shown (black triangles). To test the effect of arginine on the PpNAGK–PpPII complexes, PpPII was first added into the diluted PpNAGK solution, and the two proteins were placed at 4°C for 10 min to allow complex formation followed by the addition of arginine. The arginine effect was also tested in combination with 10 mM glutamine as described above. The continuous spectrophotometric assay was used. Values are the mean ± SD of three independent determinations. NAGK activities (100%) were **(A)** 108.80 ± 14.35, **(B)** 726.15 ± 71.58, and **(C)** 563.17 ± 29.68 nkatal/mg protein. The reaction mixtures contained **(A)** 0.65, **(B)** 0.35, and **(C)** 0.35 μg of PpNAGK protein. The observed increase of PpNAGK activity with PpPIIa and PpPIIb was significant by *t*-test with a *P* < 0.1 and enhanced in presence of glutamine with a *P* < 0.05. Arginine inhibition was significantly relieved by adding glutamine (*P* < 0.01) in both PpNAGK–PpPII complexes. Each reaction mixture contained 0.35 μg of PpNAGK protein and 0.7 μg of PpPIIa and 0.7 μg PpPIIb.

The enzyme activity of PpNAGK was increased up to aproximately fivefold when any of the two PII proteins was added in increasing concentrations (Figure 5A). Higher concentrations of PpIIa than of PpPIIb protein appeared necessary to achieved full activation of NAGK, with the maximal activation being similar for the two isoproteins. Potential differences in the interaction of PpPII isoproteins and PpNAGK were further examined by YTH analysis (Supplementary Figure S3). As depicted in Supplementary Figure S3A, the interaction was much weaker for PpPIIb-PpNAGK than for PpPIIa-PpNAGK. To study the molecular basis of this difference, we performed mutations in the PpPIIa sequence, including the change of glutamate for lysine at position 18 (Glu18Lys) and the switch of the three amino acids near the T-loop (valine, alanine, and glycine for alanine, glycine, and alanine) at positions 58, 60, and 61, respectively (Val58Ala/Ala60Gly/Gly61Ala) (Supplementary Figure S3B). Quantitative comparisons using the β-galactosidase assay confirmed the observed differences in the formation of PpPII-PpNAGK complexes and showed that these amino acid changes did not affect the interaction of PpPIIa with PpNAGK.

To further characterize the sensor properties of PpPIIa and PpPIIb toward PpNAGK, the effect of glutamine was studied in the presence of varying amounts of arginine. When the PpPIIa-PpNAGK complex was allowed to form, arginine inhibition was significantly relieved by adding glutamine (Figure 5B). Interestingly, a similar effect of glutamine was observed for the PpPIIb–PpNAGK complex (Figure 5C).

Subsequent experiments were undertaken to study whether PpNAGK activity could be affected by the interaction of both PpPIIa and PpPIIb at varying levels of 2-OG. As shown in Supplementary Figure S4, the addition of PpPIIa and PpPIIb enhanced PpNAGK activity already in the absence of glutamine. Nevertheless, under these experimental conditions, no significant differences in activity were observed at various 2-OG concentrations.

### Expression of PpPIIa and PpPIIb Genes

**FIGURE 6:**
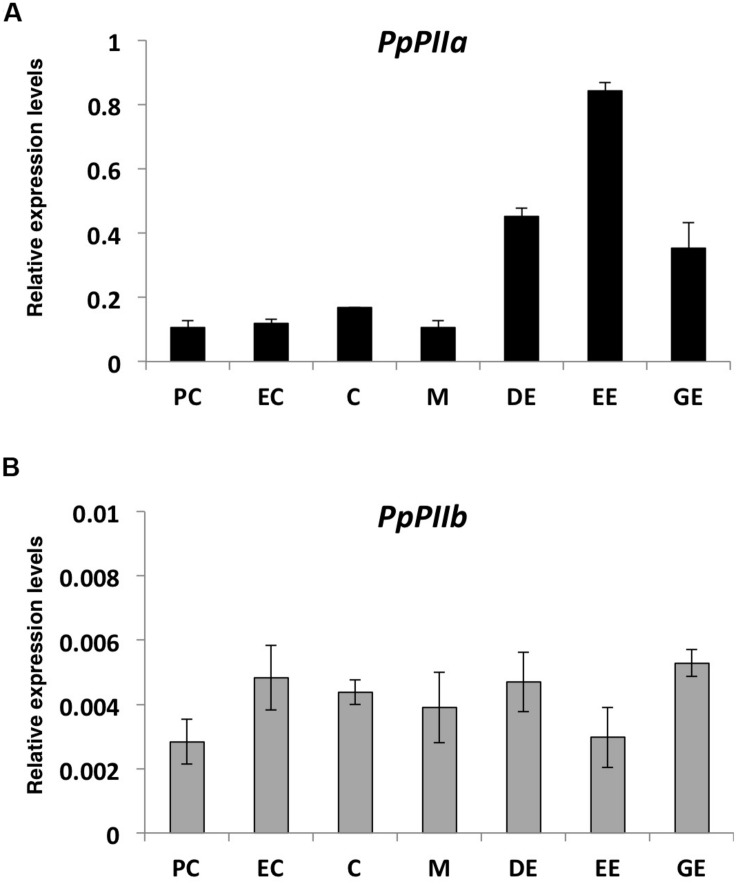
Expression analysis of pine *PII* genes during embryogenesis and early stages of seed germination **(A)** PpIIa; **(B)** PpIIb. PC, pre-cotyledonary stage; EC, early-cotyledonary; C, cotyledonary; M, cotyledonary partially mature; DE, dry embryo; EE, embedded embryo; GE, germinated embryo. The expression level for the two genes was normalized to that of *actin-7*, used as reference gene. Bars represent mean values of three assays, with three biological replicas each ± SD. Black rectangles are used for *PpPIIa gene* and gray rectangles for the *PpPIIb* gene.

To further explore the potential roles of PII isoproteins in maritime pine, gene expression levels were analyzed during the last stages of embryogenesis and seed germination (Figure 6). The relative levels of *PpPIIa* transcripts were much higher than those *PpPIIb* observed in all stages examined, with an upper abundance in the seed embryos.

## Discussion

A bioinformatics survey of the maritime pine transcriptome revealed the occurrence of two transcripts (*PpPIIa and PpPIIb*) encoding PII proteins (Supplementary Figure S1). They have similar molecular size but differ in their amino acid sequence (Figure 1). This finding is novel because only one form of PII has been reported to exist in other plant species (Chen et al., 2006). Interestingly, two PII homologs have also been identified in several pine species for which large transcriptomics data are available. In contrast, only one PII gene product has been found in spruce species (Figure 1). Taken together, our results suggest that the existence of PII variants is not a general characteristic of conifers and seems to be restricted to pines.

As the sequence of the maritime pine genome is still not available, we searched for PII homologs in the loblolly pine (*P. taeda*) genome (Neale et al., 2014). The goal was to figure out whether the two *PII* transcripts were encoded by distinct genes or they were the result of a differential splicing. The sequences for *PtPIIa* and *PtPIIb* were located in the same scaffold, but were interrupted by large gaps that prevented the retrieval of a complete gene structure.

Considering the primary structure of their cDNAs, the most plausible explanation is that PtPIIa and PtPIIb are the result of alternative splicing of a single gene. No different 3’-untranslated regions were amplified from a range of different pine tissues by rapid amplification of cDNA ends (RACE). The above conclusion is reinforced by the identification of a similar pattern of hybridizing bands in a Southern blot of pine genomic DNA digested with several restriction enzymes (Supplementary Figure S5).

Sequence analysis of the polypeptides encoded by *PtPIIa* and *PtPIIb* identified a putative cTP in their N-terminal end. Nevertheless, the existence of two forms of PII could be related with a distinct subcellular localization in pine cells. To investigate this hypothesis, we conducted subcellular localization studies (Figure 2). Our results confirmed that PpPIIa and PpPIIb are located in the chloroplastic stroma, as previously reported for their *Arabidopsis* PII homolog (Chen et al., 2006). PpPIIa and PpPIIb are therefore able to interact *in vivo* with their target enzyme, PpNAGK, which is located in the same compartment in pine cells (Llebrés et al., 2018b).

The three-dimensional models of PpPIIa and PpPIIb are highly similar to the previously described crystal structures of PII proteins from *Synechococcus* and *Arabidopsis* (Mizuno et al., 2007; Llacer et al., 2008).

These two structurally characterized PII proteins lack a glutamine site, unlike PpPII proteins, judged from sequence comparisons and from the observation of activation of NAGK activity by glutamine addition to mixtures of PpNAGK and PpPII proteins (Figures 5B,C).

The ability to sense glutamine levels, and therefore the cellular N status, relies on a C-terminal extension of the PII polypeptide that is unique of plant proteins (Chellamuthu et al., 2014). The Q loop is disordered in the absence of glutamine, but becomes ordered when the amino acid is present, which triggers in turn a specific interaction with the basal part of the T loop. The signal is then transmitted through a conformational change that favors the interaction of PII with NAGK (Chellamuthu et al., 2014; Forchhammer and Lüddecke, 2016). The change of glutamate (PpPIIa) for lysine (PpPIIb) at position 18 is spatially close to the Q loop and therefore likely to affect the above mechanism or its regulation. As shown in Figure 3, it causes a major effect on the electrostatic features of this region.

Overexpression of recombinant PpPIIa and PpPIIb in *E. coli* allowed us to confirm their trimeric nature (Figure 4), in line with previous observations for PII proteins from plants and microorganisms (Chen et al., 2006; Forchhammer and Lüddecke, 2016). Moreover, the overproduction in *E. coli* of PpNAGK, the target enzyme of PpPII, enabled us to study the formation of PpNAGK–PpPIIa and PpNAGK–PpPIIb complexes. The binding of both PpPIIa and PpPIIb to PpNAGK increased the activity of this enzyme (Figure 5). A similar observation was previously reported in *Arabidopsis* (Chen et al., 2006). PpNAGK activation suggests high affinity of PpPIIa and PpPIIb for PpNAGK in the formation of regulatory complexes, what was experimentally confirmed by YTH analysis. The sequence changes between these two proteins at positions 18 (Glu18Lys) and near the T-loop (three amino acid changes) did not affect the formation of the complex with PpNAGK, as shown by site-directed mutagenesis experiments with PpPIIa (Supplementary Figure S3). Together, the above results suggest that the observed differences between PpPIIa and PpPIIb regarding complex formation with PpNAGK are not only based in single amino acid changes.

To better characterize PpPII proteins, we also analyzed the effects of 2-OG, arginine, and glutamine on the formation of regulatory complexes with PpNAGK. The addition of increasing amounts of 2-OG did not affect PpNAGK activity when the enzyme from maritime pine was incubated in the absence of PpPIIa or PpPIIb (Supplementary Figure S4). Likewise, 2-OG did not influence PpNAGK activity when complexed with any of the two PpPII proteins, suggesting that, as in other plants having glutamine-sensing PII proteins (Chellamuthu et al., 2014), 2OG did not dissociate the complex of the two proteins. Actually, glutamine proved essential for PII-NAGK complex formation in *Chlamydomonas* and some plant species studied (excepting *A. thaliana*, which does not sense glutamine) but this appears not to be the case in maritime pine for any of it two PII proteins, although it might increase the affinity of these PpPII proteins for PpNAGK, a situation that is reminescent of that recently uncovered for the non-photoxynthetic alga *Polytomella parva* (Selim et al., 2020b).

It is well documented that the more sensitive assay for the PII-NAGK interaction is the relief of the inhibition of enzyme activity caused by arginine (Llacer et al., 2008). Chellamuthu et al. (2014) reported that glutamine is able to further relieve this feedback inhibition when NAGK is bound to PII. We have observed enhanced PpNAGK *in vitro* activity in the presence of PpPIIa and PpPIIb, when glutamine was present (Figure 5). The results suggest that PpPIIa and PpPIIb are equally effective in sensing glutamine and thus PII redundancy in pine does not appear to affect the sensing of glutamine. Alternatively, one may speculate that the occurrence of two PII variants might increase the dosage for sensing this amino acid, similarly as reported for enhanced glutamine biosynthesis in *Populus* (Castro-Rodríguez et al., 2015). Nevertheless, a differential role of PpPIIa and PpPIIb in other physiological conditions cannot be excluded.

In this regard, the expression patterns of both *PpPII* genes were analyzed at early stages of development to identify if each one may fulfill distinct roles during maritime pine embryogenesis and early growth (Figure 6). *PpPIIa* exhibited higher expression levels than *PpPIIb* suggesting its predominance, at least at these stages of development. *PpPIIa* transcripts were particularly abundant at the end of maturation and the beginning of germination when arginine metabolism is highly active. In contrast, *PpPIIb* displayed low expression levels at all stages examined (Figure 6). These results suggest different roles for these isoproteins, likely regulating in a different manner arginine metabolism through their interaction with PpNAGK.

## Conclusion

In summary, we have identified two different forms of PII in maritime pine. They are most likely the splicing products of a single gene and their existence seems to be restricted to pines: a single PII has been identified in other conifer species and in angiosperms. PpPIIa and PpPIIb are trimeric proteins with similar three-dimensional structure and subcellular location. The PII-target enzyme PpNAGK is localized to the same compartment. The interactions of both recombinant PpPII variants and PpNAGK appear to be enhanced by glutamine, relieving the feedback inhibition of PpNAGK activity by arginine in the mM range. PpIIa appears to play a predominant role during maritime pine embryogenesis and germination.

## Data Availability Statement

All datasets generated for this study are included in the article/Supplementary Material.

## Author Contributions

ML, CV, FT, JV-M, and MP performed the experimental work. ML prepared the figures. LG was in charge of protein modeling. FC and CA conceived the project, supervised the work, and wrote the manuscript with contributions from FT, MP, and LG.

## Conflict of Interest

The authors declare that the research was conducted in the absence of any commercial or financial relationships that could be construed as a potential conflict of interest.

## Footnotes

1 http://www.scbi.uma.es/sustainpinedb/sessions/new

2 www.rbvi.ucsf.edu/chimera

## Supplementary Material

FIGURE S1 Primary structure of *PpPII* full-length cDNAs. Initial and stop codons are marked in red. The position of the primers utilized for the expression analysis is underlined in red.

FIGURE S2 Purification of recombinant PpPIIa and PpPIIb proteins overproduced in *E. coli*. **(A)** Purification progress of PpPIIa. **(B)** Purification progress of PpPIIb. M, molecular makers; C, bacterial crude extract; F, flow-through eluate; W1, wash step 1; W2, wash step 2; lanes 1–5, elution of purified protein. Fractions 3 and 4 were collected for the studies performed in this work.

FIGURE S3 Yeast two-hybrid interaction analysis. **(A)** Auxotrophic analysis of interaction of PpNAGK and PpPIIa or PpPIIb. Tranformants were analyzed in SD agar plates without tryptophan, leucine, and uracil, and several dilutions were made. **(B)** Diagram of amino acid changes performed in the isoprotein PpPIIa (PpPIIa^Mut1^: Glu18Lys and PpPIIa^Mut2^: Val58Ala/Ala60Gly/Gly61Ala). **(C)** β-Galactosidase activity. Bars represent mean values of three assays, with three biological replicates each ± SD; asterisk indicates significant difference between samples at *P* < 0.05.

FIGURE S4 Effect of 2-OG concentration on PpNAGK activity. The activity of enzyme was measured in the presence of varying amounts of 2-OG, either without PpPII protein added (black triangles), or in the presence of PpPIIa (black squares) or PpPIIb (open circles) at the PpNAGK–PpPII ratios indicated. Each reaction mixture contained 0.65 μg of PpNAGK protein and 6.5 μg of PpPIIa and 6.5 μg PpPIIb. The *N*-acetylglutamylhydroxamate assay was used. Bars represent mean values of three independent experiments ± SD.

FIGURE S5 Southern blot analysis of pine genomic DNA. Isolated genomic DNA was digested with the restriction enzymes *Eco*RI, *Hind*III y *Bam*H, and hybridized with a PpPIIa cDNA probe.

TABLE S1 List of the gene specific primer sequences used in this work.

TABLE S2 Characteristics of PpPIIa and PpPIIb polypeptides and processed proteins. cTP: chloroplast transit peptide. Sequences of PpPIIa and PpPIIb were available at SustainPineDB v.3.0 (http://www.scbi.uma.es/sustainpinedb/sessions/new).
