# Supplementary material for: Structural and Functional Characteristics of Two Molecular Variants of the Nitrogen Sensor PII in Maritime Pine
Source: Front Plant Sci. 2020 Jun 16;11:823. doi: 10.3389/fpls.2020.00823 (PMC7308587; doi:10.3389/fpls.2020.00823)
Supplement: FIGURE S1 — Primary structure of PpPII full-length cDNAs. Initial and stop codons are marked in red. The position of the primers utilized for the expression analysis is underlined in red. [file Data_Sheet_1.PDF]

A)

```
P.pinaster_PIIa     1 AGTAGAATAAATACAAGTGTTGTCCGTATATTGTATCCACGTGTCATGTGACTTAGGTGGAACACCGTATGGAATGAACTCCA
P.pinaster_PIIb     1 AA--G--T-AATGCACCTCTACTCCAT---------GGAATTGTCAA----TTT--GTGA--TA-TGCATGG-----------

P.pinaster_PIIa    84 TCTTATGAAATTTTTGGTTGTGTGAGAAAATAATGCAGCTCTACTAAAGTCAGACATACTGTCAAGTCACAATGTATGATCCG
P.pinaster_PIIb    50 ---TA--------TTGGTTT--CGACGAAATGGT-CAGCAGTAGGGGAGTC-------GTGTCTCGTAACTACCGAGTAACC-

P.pinaster_PIIa   167 CAGTGTCTAGATTCCGATTGATGGTAAGCCCTCAGGGATTCAGGGATTTGTAAATCCGAGTATTGCGTAAATTAAAAGCGAAA
P.pinaster_PIIb   111 ---------GAGTAAGAGTGGCG-----CAACCAGCGAAACAAAT-TATTTATATGC---TTCTGTGTATAT--------AAA

P.pinaster_PIIa   250 TCTAAAAGGCAAAGGTACTTGTTCGCTTGTGTATATGTATAATTCTGGGCTGGTTTGTGAAAGAAATGTGTAGCGAGCTCTGC
P.pinaster_PIIb   168 TCT-------------------------GTGTA-ATGT-----GCTTGCTCGGCTTGTGAAACAAAGGAGTTTCGAGCTCTGC

P.pinaster_PIIa   333 TACCAAAGTTGCCCCGTGTTGCACACGAGTAGGGCTAGTTACCAGTAATAAACATGGCTGCCCATCTGCCTCTATTTGTCAAA
P.pinaster_PIIb   220 GGCCAAAGGTATCCGGAGTTGCGCGCGAGTGGGGCAAATTACCTAGTGTAAACATGCCTGCCCATACGCTTTTGTTTGCCAAC

P.pinaster_PIIa   416 GGATCTATATTTTCTCTACCATCATCGACATCGACGTCTTCTTCCTGTACGTATTCGAGCATATCGCATACAGCGGCATCTCC
P.pinaster_PIIb   303 GGATCTCTATTATCTCTGTCATCTT---------CATCTTCTTCTTTTACATGTTCCAGCACATGCAATGCAGCGGCATGTGC

P.pinaster_PIIa   499 TGGATTTCACTCATCTCCAGCAGCAGCCAATAAATCTGTAAAATTGGGAACGATGCAGAGGATGGATGGTGTCAGGTCGGTGA
P.pinaster_PIIb   377 TGGATTTCACTCATCTCCGGCAGCAGTCAATAATTCTGTAAAGTGGGGGGCAGTGCACAGGATGAATGGTGTGAGGTGGGTGA

P.pinaster_PIIa   582 AGCACCGAATGACAAGTCTGCATGCAAAGATGGAGAAGAGGCAACAGATTAAGGCATCGGCCCAGGCACCAAATGGGACCAAC
P.pinaster_PIIb   460 ACCACCGAATTGTAATTCTGGATGGAAAGATGAAGAAGCAGCAACAGATAAAGGCATCGGCCCAGGCACCAAATGCAACCAAC

P.pinaster_PIIa   665 ACTCATCCAGATTATGTCCCAGAAGCCAACTTTTACAAAGTAGAAGCAATATTGAGGCCATGGCGCATCTCCCATGTGACTAC
P.pinaster_PIIb   543 ACTTGTCCGGATTATATCCCGAAAGCCAATTTTTACAAAGTAGAAGCTATTTTGAGGCCATGGCGCATGTCTCATGTTAATTC

P.pinaster_PIIa   748 GGGTCTATTGAAAATGGGGATTCGTGGCGTAACTGTCTCTGATGTTAGAGGTTTTGGAGTTCAGGCTGGATCTGCAGAACGGC
P.pinaster_PIIb   626 GGGTTTATTGAAATTGGGGGTTCATGGCGTAACTGTCTCTGATGTTAAAGGTTTTGGAGCTCAGGGCGCATCTGCAGAGCGGC

P.pinaster_PIIa   831 AAGCAGGCTCCGAGTTTTCTAAAGACAATTTTGTGTCAAAAATAAAGATGGAGATTGTGGTATCTAAAGATCAGGTAGAAGCA
P.pinaster_PIIb   709 AAGCAGGCTCCGAGTTTTCTAAAGACAATTTTGTGTCAAAAGTAAAGATGGAGATTGTGGTATCTAAAGATCAGGTAGAAGCA

P.pinaster_PIIa   914 GTAATTGATGCAATCATTGATGAGGCAAGAACTGGAGAAATTGGAGATGGAAAAATATTTGTGGTTCCAGTTGCAGATGTCAT
P.pinaster_PIIb   792 GTAATTGATGCAATCATTGATGAGGCAAGAACTGGAGAAATTGGAGATGGAAAAATATTTGTGGTTCCAGTTGCAGATGTCAT

P.pinaster_PIIa   997 TCGTGTGAGAACAGGTGAGCGTGGACTTGAAGCAGAGAGAATGGCTGGTGGACGATCAGAGATACTTACAGGTGTACATCAAG
P.pinaster_PIIb   874 TCGTGTGAGAACAGGTGAGCGTGGACTTGAAGCAGAGAGAATGGCTGGTGGACGATCAGAGATACTTACAGGTGTACATCAAG

P.pinaster_PIIa  1080 AGGTTACAGACAGCAACTAAATCACTTTT-AGACACTATAA-TTGAGCCAAAGCCTGACGCTTGAGTATGGTGCACAGGAGTA
P.pinaster_PIIb   958 AGGTTACAGACAGCAACTAAATCACTTTTGAGACACTATAAATTGAGCCAAAGCCTGACGCTTGAGTATGGTGCACAGGAGTA

P.pinaster_PIIa  1161 AATAGATTTGAGATATGGTGATTGGATGGCTTTAGATGGAAGTGGTCAAACTTTATCATTTATGTTAATTGACTGTAGAGTTT
P.pinaster_PIIb  1041 AATAGATT---------------------------------------------------------------------------

P.pinaster_PIIa  1244 AAATATTAAAGATTTACGTCTCTAGAGGGGATACATTGTTCATTGTCTTGATAAAAATAGATTATCTTATTCTGTAATATTGT
P.pinaster_PIIb  1033 -----------------------------------------------------------------------------------

P.pinaster_PIIa  1327 TCAGTTGTGGCTATTAGAATTTGGATCTTTTGAAGTTGAAATACATGCTATCAACATTTTCATTTCCAATAATATTTTGTCAG
P.pinaster_PIIb       -----------------------------------------------------------------------------------

P.pinaster_PIIa  1410 CTTTGGCAATAACAATCTGAAATTGTAGGAATGTTTGAAAAATAAGGTTGTCTCTGCAGACACACGTACACAGATCAGTAGTA
P.pinaster_PIIb       -----------------------------------------------------------------------------------

P.pinaster_PIIa  1493 GAGGGGAAAAGCTCTTATTGGCAGTGAGTAAATATCCAATGACACATGATTGTTGTTGGTCCTATATGGTACGGGGTTTGTAT
P.pinaster_PIIb       -----------------------------------------------------------------------------------

P.pinaster_PIIa  1576 TGGCTGTAGATACACGGAATTGTAGTGCACCCAAGCATTCAGAAGCATCGGGGCCCATCTTTTGAGGGCCTTCTCTCACAAAA
P.pinaster_PIIb       -----------------------------------------------------------------------------------

P.pinaster_PIIa  1659 TATTTGGTCCGCTATATATTTAAGCTAAAACGAGTTACTACTTTTAATAAAAATTTCGATGGGGAGTACACAACATGTATCAA
P.pinaster_PIIb       -----------------------------------------------------------------------------------

P.pinaster_PIIa  1742 GCATCAATTGAGATGGGAACCTGCAGACAAGCAGCTTGATACTCACAATCACGGAATATTGACAATAAAATGTAGAATTTTGT
P.pinaster_PIIb       -----------------------------------------------------------------------------------

P.pinaster_PIIa  1825 TGGGTGGAGTTGTCCAAAGGGCTGGAGAACCTAAGGATTTCCTCGGGGATTAGCTCTCCATATTGTGTGACAATGCATCATCT
P.pinaster_PIIb       -----------------------------------------------------------------------------------

P.pinaster_PIIa  1908 TATTGATTTTTGAGCTAAAGGCAATTTAGTGAAGCTCCATTGTGTACATACCATCTACCTCGAAG
P.pinaster_PIIb       -----------------------------------------------------------------
```
