# Supplementary figures and images for: Structural and Functional Characteristics of Two Molecular Variants of the Nitrogen Sensor PII in Maritime Pine
Source: Front Plant Sci. 2020 Jun 16;11:823. doi: 10.3389/fpls.2020.00823 (PMC7308587; doi:10.3389/fpls.2020.00823)

A)

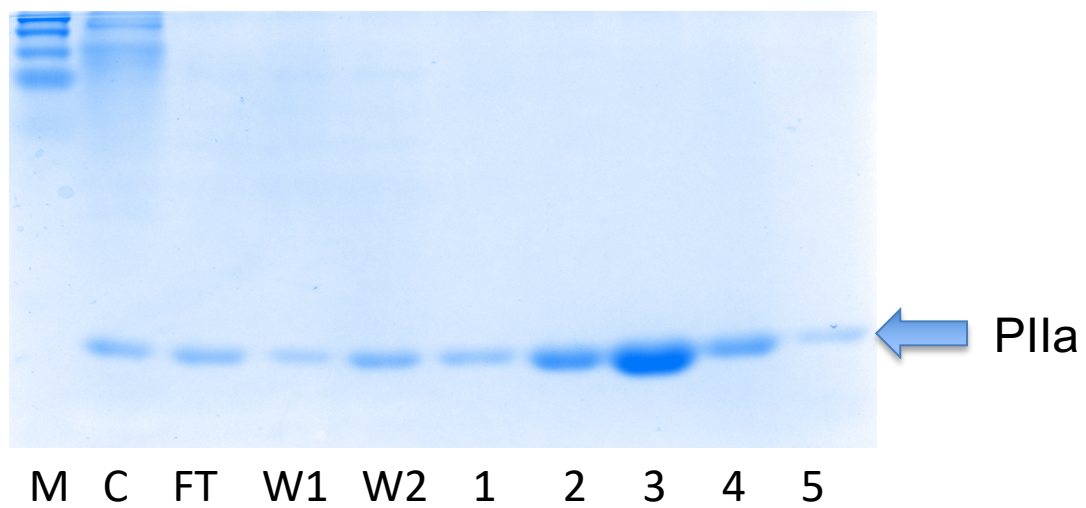

B)

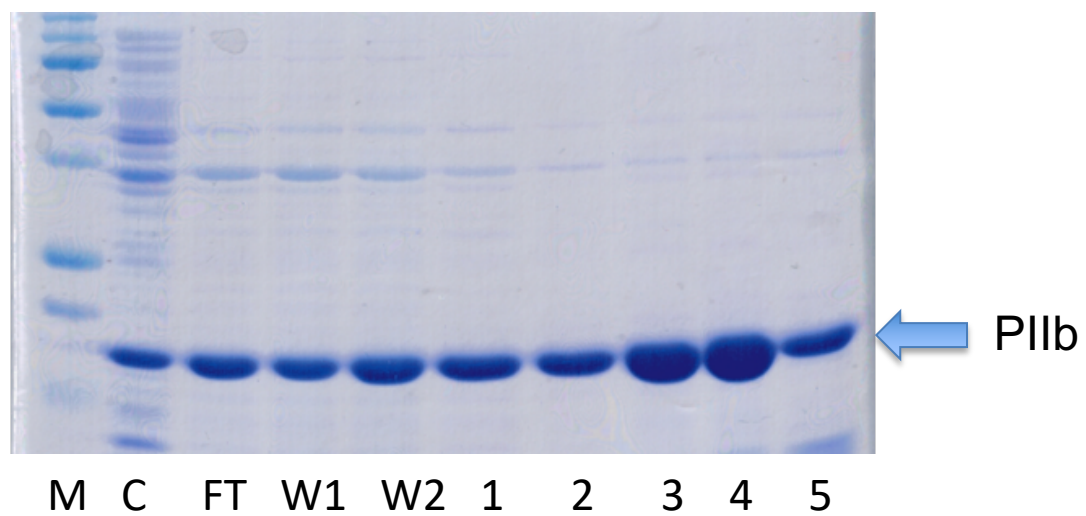

Supplement: FIGURE S2 — Purification of recombinant PpPIIa and PpPIIb proteins overproduced in E. coli. (A) Purification progress of PpPIIa. (B) Purification progress of PpPIIb. M, molecular makers; C, bacterial crude extract; F, flow-through eluate; W1, wash step 1; W2, wash step 2; lanes 1–5, elution of purified protein. Fractions 3 and 4 were collected for the studies performed in this work. [file Data_Sheet_2.PDF]

A)

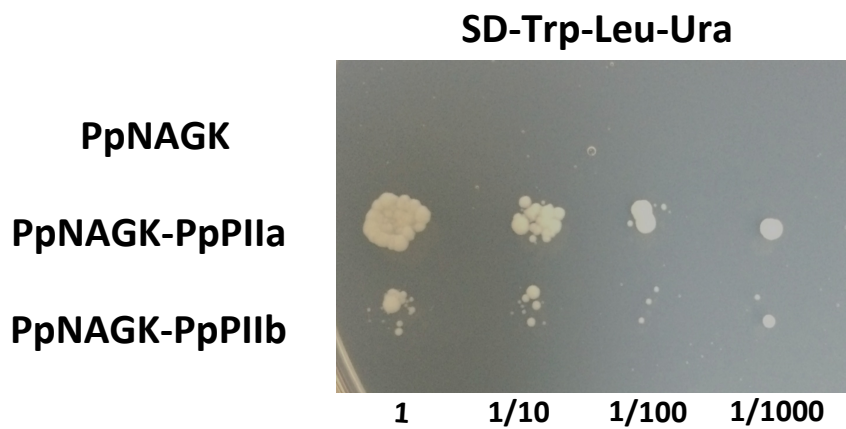

B)

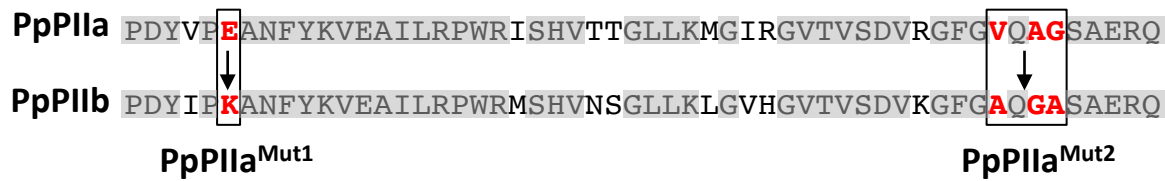

C)

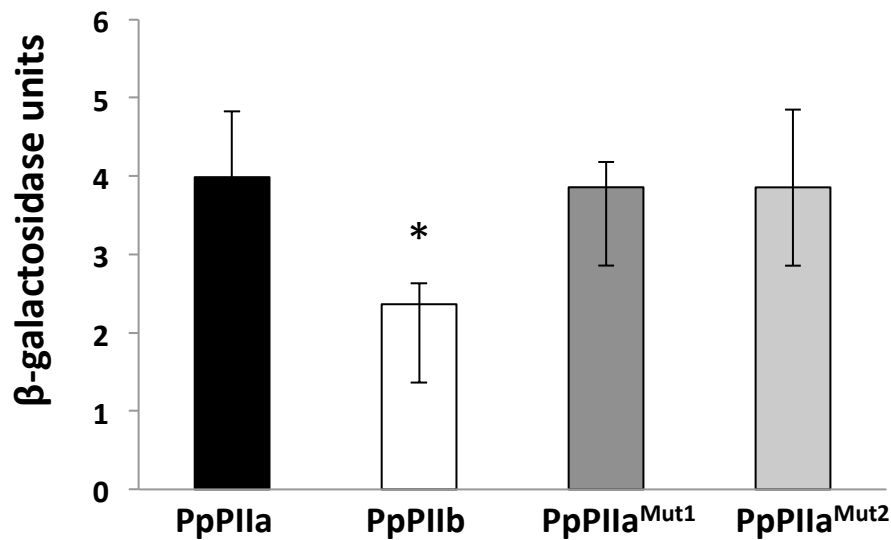

Supplement: FIGURE S3 — Yeast two-hybrid interaction analysis. (A) Auxotrophic analysis of interaction of PpNAGK and PpPIIa or PpPIIb. Tranformants were analyzed in SD agar plates without tryptophan, leucine, and uracil, and several dilutions were made. (B) Diagram of amino acid changes performed in the isoprotein PpPIIa (PpPIIaMut1: Glu18Lys and PpPIIaMut2: Val58Ala/Ala60Gly/Gly61Ala). (C) β-Galactosidase activity. Bars represent mean values of three assays, with three biological replicates each ± SD; asterisk indicates significant difference between samples at P < 0.05. [file Data_Sheet_3.PDF]

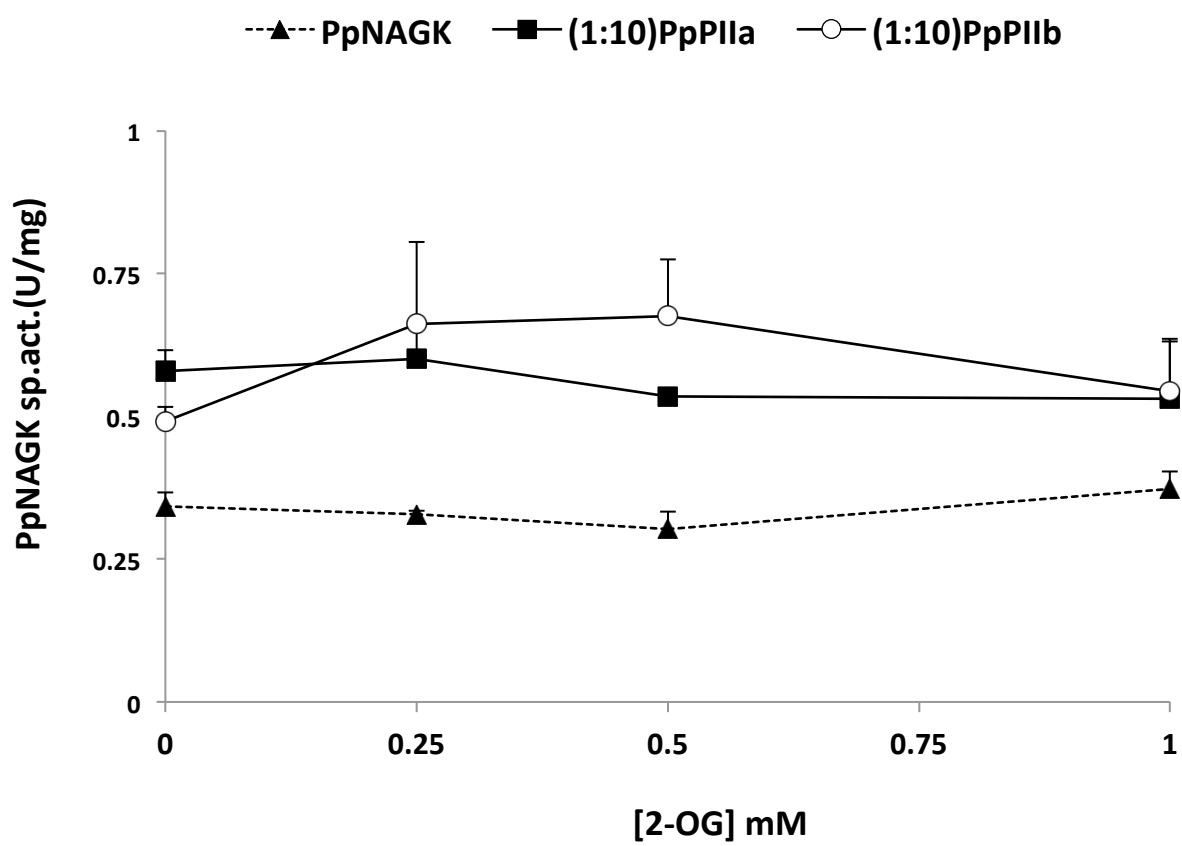

Supplement: FIGURE S4 — Effect of 2-OG concentration on PpNAGK activity. The activity of enzyme was measured in the presence of varying amounts of 2-OG, either without PpPII protein added (black triangles), or in the presence of PpPIIa (black squares) or PpPIIb (open circles) at the PpNAGK–PpPII ratios indicated. Each reaction mixture contained 0.65 μg of PpNAGK protein and 6.5 μg of PpPIIa and 6.5 μg PpPIIb. The N-acetylglutamylhydroxamate assay was used. Bars represent mean values of three independent experiments ± SD. [file Data_Sheet_4.PDF]

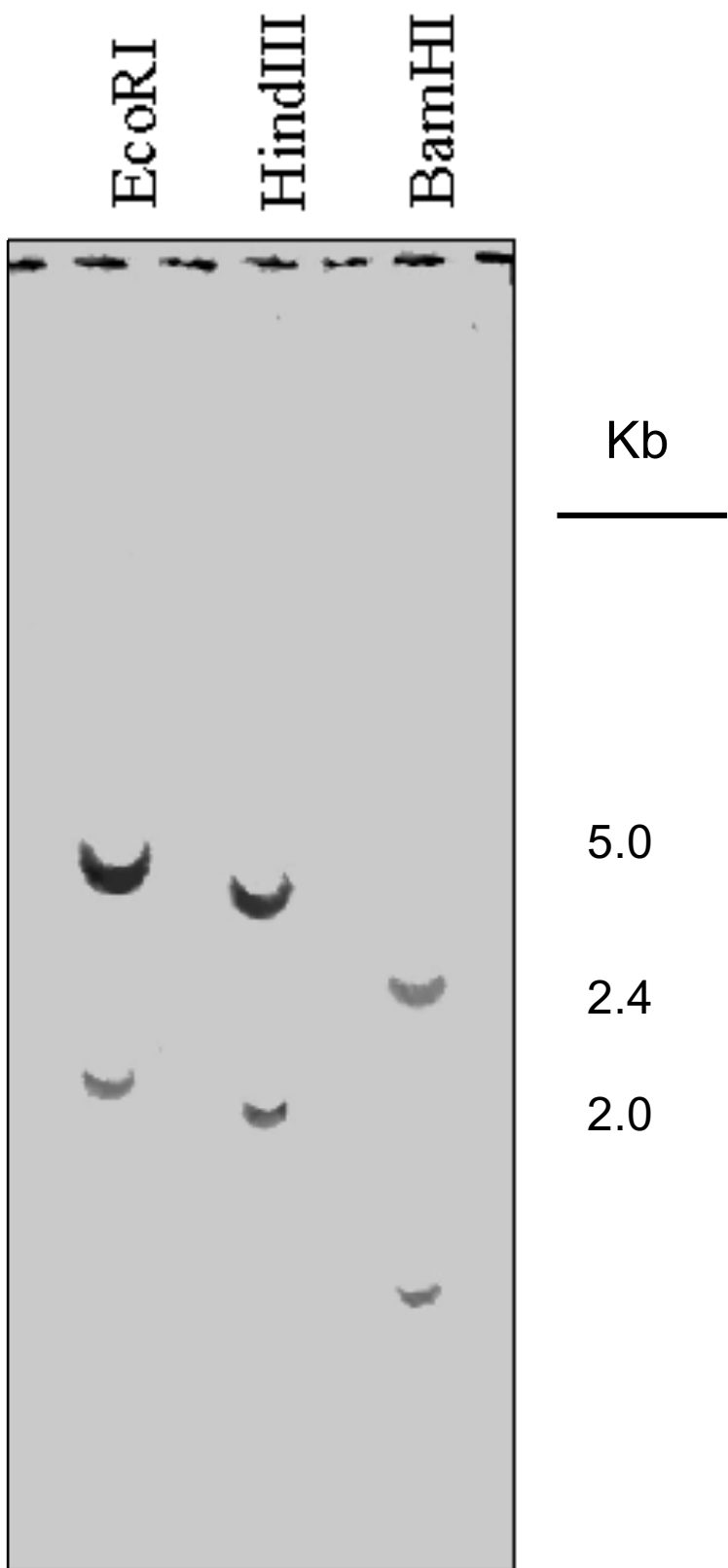

Supplement: FIGURE S5 — Southern blot analysis of pine genomic DNA. Isolated genomic DNA was digested with the restriction enzymes EcoRI, HindIII y BamH, and hybridized with a PpPIIa cDNA probe. [file Data_Sheet_5.PDF]
