# Supplementary material for: Structural and Functional Characteristics of Two Molecular Variants of the Nitrogen Sensor PII in Maritime Pine
Source: Front Plant Sci. 2020 Jun 16;11:823. doi: 10.3389/fpls.2020.00823 (PMC7308587; doi:10.3389/fpls.2020.00823)
Supplement: TABLE S1 — List of the gene specific primer sequences used in this work. [file Table_1.DOCX]

**Supplemental material**

**Table S1. List of the gene specific primer sequences used in this work.**

| **Name** | **Sequence** |
| --- | --- |
| **Cloning** | |
| PpPIIa_Fwd | 5´-AAAAAGCAGGCTTAATGGCTGCCCATCTGCCTC-3´ |
| PpPIIb_Fwd | 5´-AAAAAGCAGGCTTAATGCCTGCCCATACGCTTT-3´ |
| PpPIIab_Rev | 5´-GAAAGCTGGGTCTTAGTTGCTGTCTGTAACC-3´ |
| PpNAGK_Fwd | 5´-AAAGCAGGCTTCATGCGGGCGGCTTCAATG-3´ |
| PpNAGK_Rev | 5´-GAAAGCTGGGTCTCAACCTGTGATCATCGTTC-3´ |
| PpPIIab_Rev-stop | 5´-AGAAAGCTGGGTCGTTGCTGTCTGTAACCTC-3´ |
| PpNAGK_Rev-stop | 5´-AGAAAGCTGGGTCACCTGTGATCATCGTTCCAG-3´ |
| PpPIIa-cTP_Fwd | 5´-AAAGCAGGCTTACAGGCACCAAATGGGACC-3´ |
| PpPIIb-cTP_Fwd | 5´-AAAGCAGGCTTACAGGCACCAAATGCAACC-3´ |
| **Overexpression** | |
| PpPIIa-cTPXhoI_Fwd | 5´-CGCATATGCAGGCACCAAATGGGACC-3´ |
| PpPIIb-cTPXhoI_Fwd | 5´-CGCATATGCAGGCACCAAATGCAACC-3´ |
| PpPIIab-NdeI_Rev | 5´-GGCTCGAGGTTGCTGTCTGTAACCTC-3´ |
| PpNAGK-cTP_Fwd | 5´-AAAAAGCAGGCTTAAGGAAATCCAGAGGCGCCC-3´ |
| **Expression analysis** | |
| PpPIIa_qPCR-Fwd | 5´-TCTACCATCATCGACATCGACGTC-3´ |
| PpPIIa_qPCR-Rev | 5´-CCATCCTCTGCATCGTTCCCAAT-3´ |
| PpPIIb_qPCR-Fwd | 5´-CGGATCTCTATTATCTCTGTCATCTTCA-3´ |
| PpPIIb_qPCR-Rev | 5´-TCATCCTGTGCACTGCCCCCCAC-3´ |
| Actin7_qPCR-Fwd | 5 ́-ATCTCTCAGCACATTCCAACAG-3 ́ |
| Actin7_qPCR-Rev | 5 ́-TATTGCCACCATCATCTCAAGC-3 ́ |
