# Supplementary material for: Structural and Functional Characteristics of Two Molecular Variants of the Nitrogen Sensor PII in Maritime Pine
Source: Front Plant Sci. 2020 Jun 16;11:823. doi: 10.3389/fpls.2020.00823 (PMC7308587; doi:10.3389/fpls.2020.00823)
Supplement: TABLE S2 — Characteristics of PpPIIa and PpPIIb polypeptides and processed proteins. cTP: chloroplast transit peptide. Sequences of PpPIIa and PpPIIb were available at SustainPineDB v.3.0 (http://www.scbi.uma.es/sustainpinedb/sessions/new). [file Table_2.DOCX]

**Table S2. Characteristics of PpPIIa and PpPIIb polypeptides.** cTP: Chloroplast transit peptide.

|  | **PpPIIa** | **PpPIIa-cTP** | **PpPIIb** | **PpPIIb-cTP** |
| --- | --- | --- | --- | --- |
| **Molecular size (Da)** | 25,467 | 16,579 | 25,009 | 16,482 |
| **Amino acids** | 237 | 154 | 234 | 154 |
| **Isoelectric point** | 9.38 | 5.42 | 8.91 | 5.64 |
